# A minimally invasive marine mammal sex determination method using epidermal tissue recovered from suction-cup tags

**DOI:** 10.1371/journal.pone.0323658

**Published:** 2025-05-23

**Authors:** Sadie X. Novak, Jacob M.J. Linsky, Kaitlyn J. Knapp, Susan E. Parks, David Wiley, Dana A. Cusano

**Affiliations:** 1 Chemistry Department, Syracuse University, Syracuse, New York, United States of America; 2 Moreton Bay Research Station, The University of Queensland, St. Lucia, Queensland, Australia; 3 Department of Biology, Syracuse University, Syracuse, New York, United States of America; 4 Stellwagen Bank National Marine Sanctuary, Scituate, Massachusetts, United States of America; University of Maryland Center for Environmental Science, UNITED STATES OF AMERICA

## Abstract

For many baleen whales, minimal sexual dimorphism means that sex cannot necessarily be reliably determined through observation alone. Suction cup tags deployed for behavioral studies of free-ranging whales sometimes retain exfoliated epidermal tissue from the tagged animal that can potentially be used for molecular genetic sexing. This study provides a protocol to recover and preserve skin from suction cup tags and compares the accuracy of resulting PCR-based sex determinations relative to independent data on individual sex. Skin samples (N = 43) were recovered from tags deployed on North Atlantic humpback and right whales of known sex. Of these, data were obtained from 21 tags deployed on 15 individuals. A PCR-based sex determination yielded correct results for 9 male and 11 female samples. One female was misidentified as male in one of two samples collected from the same tag, suggesting the potential for sexing error based on suction cup tag-derived tissue. The probability of accurate results given current information was estimated using Bayes theorem, with a female result estimated to be 100% reliable, and a male result estimated to be 91% reliable. The minimum DNA yield that resulted in a successful PCR run was 29 ng, however four samples with higher DNA yield did not. With further work, this protocol may increase the reliable data that can be collected as part of suction-cup tagging studies of cetaceans.

## Introduction

Behavioral differences between males and females are important to understand for ecological research and species management. As one example, sex-based differences in prey preference and social behavior can lead to disparate spatial distributions of males vs. females (e.g., sperm whales, *Physeter macrocephalus* [1]) making knowledge of an individual’s sex critical in population research and conservation [2]. This is especially true for endangered species for which data on individual sex is often limited.

Methods of determining the sex of cetaceans (whales, dolphins, and porpoises) are generally limited to observation and remote sampling methods (e.g., biopsy sampling). Observational data includes sexing females by the presence of a dependent calf, for either sex, or by visually documenting sex-based differences in external morphology [3]. While sometimes one or both of these methods is sufficient for sex identification, they can present challenges, particularly for whales that typically have few distinct or consistently visible morphological differences between sexes. As one example, females and immature males are difficult to differentiate visually in northern bottlenose whales (*Hyperoodon ampullatus*) [4]). In baleen whales, although there is evidence of reversed sexual dimorphism (i.e., females are larger than males), the size difference is relatively small (5%) [5]. Further, in species such as the humpback whale (*Megaptera novaeangliae*), estimating total body length from observational data using partial body length is problematic due to the high variability in this ratio [6].

Remote sampling methods combined with genetic techniques have enabled researchers an opportunity to circumvent the challenges presented with observational methods, and are well-established for determining sex using DNA collected from materials such as skin, breath mucosa, and excrement [7]. In cetaceans, skin samples are the most widely used and are typically collected via minimally-invasive, remote biopsy sampling methods [8]. Naturally sloughed skin is an alternative, opportunistic method of obtaining skin and has also been shown to successfully yield DNA in cetaceans (e.g., humpback whale [9–11]; sperm whale [11,12], North Atlantic right whale, *Eubalaena glacialis* [11,13]). Sloughed skin can be readily available in the water following surface activity like breaching [10] or during social interactions between conspecifics [12]. However, when multiple individuals are associated or in close proximity, those samples can be difficult to link to specific individuals.

Minimally invasive suction cup tags are widely used to study cetacean behavior. As the suction cups attach directly to the animal, they can retain skin tissue that can be an opportunistic source of DNA material upon tag retrieval, potentially allowing for sex determination and other analyses at the individual level [14], even with samples that may not be visible to the naked eye. Previously, this method has been used to determine the sex of blue whales (*Balaenoptera musculus* [15]) and sei whales (*Balaenoptera borealis* [16]), however these data were not suitable for ground-truthing this technique as the sex of the individual was unknown. Further, there is no established protocol for tag-based sample collection, limiting the robust understanding of potential error for studies that adopt this technique.

The goal of the present study was to assess cetacean sex determination from skin samples obtained from suction-cup deployed tags. We hypothesized that the suction cups may carry trace amounts of sloughed skin viable for DNA extraction. In this study, we extracted DNA collected from tags deployed on two baleen whale species. To validate the use of tissue collected in this manner, this study collected samples from suction cups of tags deployed on humpback whales in the Gulf of Maine and North Atlantic right whales. These populations have been studied extensively over several decades and many of the individuals are of known sex [17,18]. The methodology and validations outlined in this study aim to encourage a more widespread application of this non-invasive sampling technique.

## Materials and methods

The protocol described in this article is published on *protocols*.*io*, **DOI: dx.doi.org/10.17504/protocols.io.5qpvor42dv4o/v1** and is included as a supporting information S1 Appendix.

### Field data collection

Data from North Atlantic humpback whales were collected off the coast of Massachusetts, in and around the Stellwagen Bank National Marine Sanctuary in the southern Gulf of Maine in July/August 2022 and July 2023. As part of a long-term behavioral study, humpback whales were tagged with suction-cup acoustic recording tags (DTAGs [19]; CATS tags [20]) that remained attached for up to ~33 hours (minimum ~5 minutes). Additionally, data from North Atlantic right whales were collected in southern New England in May 2023 and in Cape Cod Bay in March 2024 using the same tag platforms and methodology. Tags on North Atlantic right whales remained attached for up to ~30 hours (minimum ~30 minutes). Tag deployments were approved by the Institutional Animal Care and Use Committee of Syracuse University (IACUC approval numbers 20-002 and 23-002).

Tags were adrift in the ocean for various time periods before retrieval (~5 minutes – ~ 18 hours). Upon retrieval of the tag, suction-cups were wiped with sterile foam-tipped swabs (Puritan, cat. no. 25-1506 1PF) regardless of visible skin. During later stages of the protocol development, two swabs per tag were collected (one from the front two suction cups and one from the back two suction cups) to provide additional data for validation. The swabs were then placed into sample vials, labelled with the deployment details, and immediately moved to a -20 freezer located onboard the vessel. Upon returning to land, samples were stored in a -80°C freezer.

### DNA extraction methodologies

Genomic DNA was extracted from collected tissue samples using the MyTaq Extract-PCR kit (Meridian Bioscience, cat. no. BIO-21126) or the DNeasy Blood and Tissue Kit (Qiagen, cat. no. 69504) following the manufacturer protocols. Briefly, the swab tip of the collection swab was detached from the applicator shaft using a clean razor blade. The swab tip was returned to the original storage vial and DNA extraction was performed. DNA extraction using the MyTaq Extract-PCR kit followed the manufacturer’s standard protocol. DNA extraction using the DNeasy Blood and Tissue Kit was performed following the manufacturer’s standard protocol, with the following exceptions: 1) during lysis, samples were incubated with the provided lysis buffer and proteinase K solution for 3 hours in a hot water bath at 37°C; 2) tissue lysate was incubated on the DNA-binding spin column membrane for 5 minutes to allow DNA to bind before centrifuging; and 3) purified DNA was eluted from the DNA-binding spin column in 100 μL of Elution Buffer two times, to yield a dilute solution of purified DNA at a final volume of 200 µL. Samples were then dried under vacuum for 10 hours at ambient temperature using a Vacufuge Plus vacuum centrifuge (Eppendorf, Hamburg, Germany). The resulting DNA sample was re-solubilized in 20 µL of ultrapure water.

The DNA yield (ng/µL) and sample purity (A260/A280 value) were measured using a Nanodrop Spectrophotometer 2000/2000c (Thermo Fisher Scientific, Wilmington, NC). Samples with an A260/A280 value of 1.8–2.0 were considered pure and continued to PCR analysis. The DNA samples extracted using the MyTaq Extract-PCR kit did not undergo pre-amplification screening for yield and purity. Samples of extracted DNA were stored at -20°C until downstream analysis.

### Primers

This study used primer sequences specifically targeting the homologous ZFX/XFY (zinc-finger protein genes located on both X and Y chromosomes) regions and specific oligonucleotide primer sequences targeting the Y-linked sex-determining region Y (SRY) that were previously designed and validated for sex determination of cetaceans and dugongs [21]. Lyophilized oligonucleotide primers targeting the SRY gene (forward: CCC ATG AAC GCA TTC ATT GTG TGG; reverse: ATT TTA GCC TTC CGA CGA GGT CGA TA) and ZFX/ZFY (forward: ATA ATC ACA TGG AGA GCC ACA AGC T; reverse: GCA CTT CTT TGG TAT CTG AGA AAG T) were synthesized by Integrated DNA Technologies (Coralville, IA). The oligonucleotides were re-solubilized in ultrapure water to a final concentration of 100 uM and stored at -20°C.

### PCR

PCR reactions were carried out in 8-tube strips of 0.2-mL PCR-grade reaction tubes in a final reaction volume of 25 µL containing 1X OneTaq Standard Reaction Buffer (New England Biolabs, cat. no. B9022SVIAL), 0.4 µM primers (ZFX/ZFY forward and reverse and SRY forward and reverse primers), 200 µM dNTPs (Thermo Fisher Scientific, cat. no. 18427013), 2–5 µL extracted DNA, and 2.5 U OneTaq DNA polymerase (New England Biolabs, cat. no. M0480S) in deionized water. The PCR amplification was performed using a MyCycler thermal cycler (Bio-Rad, Hercules, CA). Samples underwent 32 total cycles of PCR amplification at the following conditions: 1 cycle of initial denaturation at 95°C for 180 seconds, 30 cycles of denaturation at 95°C for 15 seconds, annealing at 55°C for 15 seconds, extension at 72°C for 20 seconds, and 1 cycle for a final extension at 72°C for 300 seconds. Reaction products were resolved by electrophoresis in a 2% agarose gel stained with 1X SYBR Safe DNA gel stain (Invitrogen, cat. no. S33102). An amplicon was expected at 210–224 bp for the presence of the SRY gene. An amplicon at 442–445 was expected for the presence of the ZFX/ZFY gene.

Presence of amplicons for both genes indicated analyzed DNA was isolated from a male individual, whereas absence of the amplicon for the Y-linked SRY gene indicated analyzed DNA was isolated from a female individual, as females do not carry the chromosome (although absence can also indicate amplification failure – see below). Because this test is based upon the absence of the SRY gene, inclusion of primers targeting ZFX/ZFY gene regions also served as an internal reference control for amplification and viable DNA presence for analysis [21,22].

### Assessing protocol performance

#### Validation data.

All tagged individuals of both species had been cataloged by the Gulf of Maine Humpback Whale Catalog [18] or the North Atlantic Right Whale Catalog [17] which curate data on the sex of the catalogued individuals. For both species, sexes were based on molecular genetic sexing of skin from biopsy samples [22]. Biopsy samples for humpback whales were collected as part of long-term population studies by the Center for Coastal Studies (Provincetown, MA), and sexes were determined by the Marine Evolution and Conservation Group, Groningen Institute of Evolutionary Life Sciences, University of Groningen (Netherlands), as described by [22]. There was often additional, supporting data on sex from observational methods for studied individuals [23,24]. Biopsy samples for North Atlantic right whales were also collected as part of long-term population monitoring by the Anderson Cabot Center for Ocean Life at the New England Aquarium (Boston, Massachusetts), and sexes were determined by the Frasier Lab at St. Mary’s University (Halifax, Nova Scotia).

#### Validation assessment method.

The probability of an accurate test result was calculated by comparing test results to the known sex of individuals. This was accomplished for all known-sex samples that met extraction criteria, and produced visible amplification of the ZFX/ZFY control gene in gels, using an application of Bayes’ Theorem similar to assessing the diagnostic accuracy of disease testing [25]. A true positive test represents the PCR detection of a Y chromosome for a known male individual, and a true negative test when the Y chromosome is absent for a known female.

Test performance was calculated such that:


$$\frac{P(TS)*P_{o}(S)}{P(TS)*P_{o}(S)+P()*P_{o}()}$$ (1)

Where P(TS) is the probability of a true sex determination result (for males, the odds of a true positive and for females the odds of a true negative), P_o_(S) is the probability of the sex being assessed occurring in the dataset (i.e., proportion of individuals of a sex that that met protocol criteria divided by the total number of individuals assessed), P(~TS) represents the odds of a false result (false negative for males and false positive for females), and P_o_(~S) represents the probability of the opposite sex occurring in the dataset.

As a Bayesian methodology, the probabilities of a test result correctly determining the sex of an individual are intended to be updated with new information [25]. To refine our results, data collected from deployments on known-sex individuals in future field seasons will be used to update the probabilities in the associated protocol for this publication. This will allow robust information on the reliability of this method with increasing sample sizes and future protocol improvements.

## Results and discussion

A total of 43 skin samples were obtained (n = 31 humpback whales, n = 12 North Atlantic right whales). Of these, sex determination was possible for 21 samples from 15 unique individuals (Table 1). The MyTaq Extract-PCR kit yielded 9 results while the DNEasy kit yielded 12 results. A full summary of all samples can be found in the S1 Table.

**Table 1:** Summary of the samples used in the study and the results of the PCR test for sex determination. Where two samples were collected from the same deployment, separate samples were collected from the front suction cups and from the back suction cups. The sample that did not match the known sex of the individual is in bold. NARW: North Atlantic right whale.

Date	Species	Deployment ID	Verified Sex	PCR Sex Result
7/17/2022	Humpback	mn22_198a	F	F
7/17/2022	Humpback	mn22_198c	M	M
7/20/2022	Humpback	mn22_201f	F	F
5/14/2023	NARW	eg23_134b (Sample 1)	M	M
5/14/2023	NARW	eg23_134b (Sample 2)	M	M
7/17/2023	Humpback	mn23_198a (Sample 1)	F	F
7/17/2023	Humpback	mn23_198a (Sample 2)	F	F
7/18/2023	Humpback	mn23_199c (Sample 1)	F	F
**7/18/2023**	**Humpback**	**mn23_199c (Sample 2)**	**F**	**M**
7/19/2023	Humpback	mn23_200a	F	F
7/19/2023	Humpback	mn23_200c	F	F
7/22/2023	Humpback	mn23_203a	M	M
7/23/2023	Humpback	mn23_204a	M	M
7/23/2023	Humpback	mn23_204b	F	F
7/23/2023	Humpback	mn23_204c	F	F
7/25/2023	Humpback	mn23_206a	F	F
7/25/2023	Humpback	mn23_206b	M	M
7/25/2023	Humpback	mn23_206d	M	M
3/18/2024	NARW	eg24_078b	F	F
3/27/2024	NARW	eg24_087a (Sample 1)	M	M
3/27/2024	NARW	eg24_087a (Sample 2)	M	M

**Fig 1:**
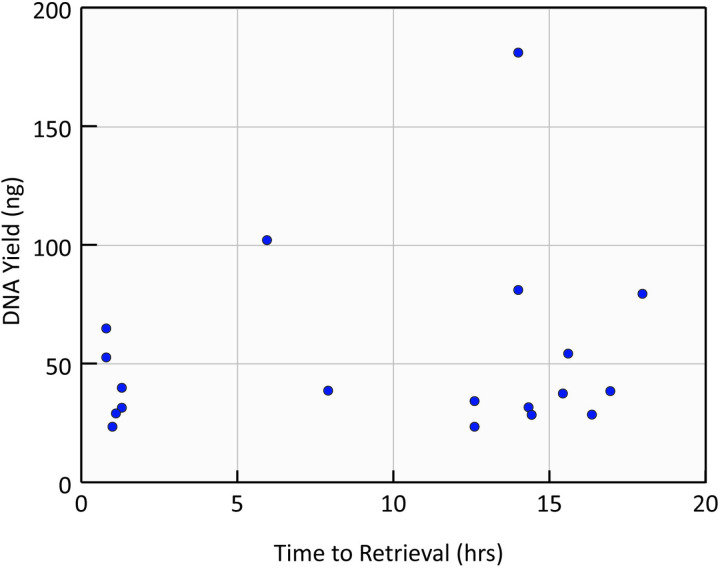
Scatterplot demonstrating the lack of a clear trend between the time the tag was adrift in the ocean (time to retrieval) and the DNA yield.

The extracted DNA yield was measured for 19 samples, twelve of which resulted in successful sex determination. With one exception, sex determination results were reliably obtained from suction cups with a DNA yield over 35 ng (Table 2). Lower yields may have resulted from a lack of skin recovered from the suction cups, as often the swab had no visible skin or particulates. There was no obvious trend between the time to retrieval and the amount of DNA yield (Fig 1), meaning the tags that drifted longer before retrieval did not have samples that contained less tissue than those that were retrieved sooner. As the amount of skin that will be available for collection is thus unpredictable, using alternative techniques that increase the DNA yield or that require less DNA should be explored.

**Table 2:** Summary of DNA yield and time to tag retrieval after the tag detached from the whale for samples where DNA yield was measured. Samples with successful PCR runs are in bold. EGNO: North Atlantic right whale individual identification number.

Date	Species	Deployment ID	PCR Results	Time to Retrieval (hrs)	DNA Yield (ng)
5/10/2023	NARW	eg23_130a	N	1.3	31.4
5/14/2023	NARW	eg23_134a (Sample 1)	N	12.6	34.2
5/14/2023	NARW	eg23_134a (Sample 2)	N	12.6	23.4
5/14/2023	NARW	**eg23_134b (Sample 1)**	**Y**	**0.8**	**52.6**
5/14/2023	NARW	**eg23_134b (Sample 2)**	**Y**	**0.8**	**64.8**
5/26/2023	NARW	eg23_146a	N	1	23.4
7/20/2023	Humpback	mn23_201a	N	14.3	31.6
7/20/2023	Humpback	mn23_201c	N	14.4	28.4
**7/22/2023**	Humpback	**mn23_203a**	**Y**	**17.9**	**79.4**
7/22/2023	Humpback	mn23_203b (Sample 1)	N	16.9	38.4
**7/23/2023**	Humpback	**mn23_204a**	**Y**	**1.3**	**39.8**
**7/23/2023**	Humpback	**mn23_204b**	**Y**	**15.4**	**37.4**
**7/23/2023**	Humpback	**mn23_204c**	**Y**	**15.6**	**54.2**
**7/23/2023**	Humpback	**mn23_206d**	**Y**	**1.1**	**29.0**
**7/23/2023**	Humpback	**mn23_206a**	**Y**	**7.9**	**38.6**
**7/23/2023**	Humpback	**mn23_206b**	**Y**	**5.9**	**102.0**
**3/18/2024**	NARW	**eg24_078b**	**Y**	**16.4**	**28.5**
**3/27/2024**	NARW	**eg24_087a (Sample 1)**	**Y**	**14**	**181.0**
**3/27/2024**	NARW	**eg24_087a (Sample 2)**	**Y**	**14**	**81.0**

### Validation

Of the 21 samples that yielded results, all but one matched the known sex of the individual whose sex was validated by genetic analysis of a tissue biopsy, as described above (Table 1). In that case, a female was misidentified as a male according to the PCR results. The misidentified sample was one of two that originated from the same tag deployment, and the other sample matched the known sex, highlighting the importance of taking multiple samples from the same tag deployment to assess the potential for error.

The probability of a true positive in the dataset (e.g., the individual was a known male and the SRY gene was present) was 1.0, while the probability of a false negative (e.g., the individual was a known male and the SRY gene was absent) was 0. The probability of obtaining a true negative in the validation dataset (e.g., the individual was a known female and the SRY gene was absent) was 0.92, whereas the probability of obtaining a false positive (e.g., the individual was a known female and the SRY gene was detected) was 0.08. Based on Bayes’ formula (Equation 1), the probability that a sample will be sexed as male when the known sex is male was 0.91 given the true sex ratio of 0.4 males to 0.5 females in this dataset. In other words, the chance of an accurate test result in the present study for males was 91%. Female protocol was found to be 100% accurate in the current dataset.

There are some opportunities in this protocol to introduce contamination which may have produced the single misidentified sample, including during the handling of the tag upon retrieval. Additionally, after release from a whale, the tag suction cups may have been exposed to whale DNA in the environment (eDNA). The risk of other animal tissue contacting and thus contaminating the suction cups is harder to mitigate, particularly in large groups or during social interactions. Care should be taken to only use samples taken from the area in direct contact with the animal (i.e., the inside of the suction cups) to reduce this risk.

Despite these potential issues, the contamination of samples when working with sloughed skin is thought to be minimal [9]. It is possible that the incorrect sex determination was instead the result of an error in the lab process, such as mishandling or amplification/read errors. Further refinements to this protocol could improve the efficiency of DNA extraction and the accuracy of results, including the use of more specific primers. Future studies on the efficacy of the provided protocol should include an analysis of additional environmental impacts on sample viability considering salinity, sun exposure, and time may all have an impact on DNA retention. Lastly, more detailed validation of tag-based results against other data (e.g., mtDNA) would be beneficial.

## Conclusions

This study develops an initial protocol to collect cetacean epidermal tissue from suction-cup tags for the purpose of molecular genetic sexing. Although qPCR methods can be used to determine the sex of cetaceans [26], and may be a more sensitive method if the equipment is available, the protocol here outlines a cost effective method that may expand the opportunities to sex an individual during tag-based studies. This study also offers a way to quantify error using an application of Bayes theorem that is similar to the assessment of medical tests, resulting in the ability to determine a 100% protocol accuracy rate for females and a 91% accuracy rate for males. By continuing to collect data and update these probabilities with larger sample sizes, the performance of this protocol can be assessed over time. Future studies can access the most up to date figures in the published protocol to decide whether this method is sufficient for their intended margin of error. Future research should also focus on refining this method to increase DNA yield from suction cups and improve the accuracy of sex determination. Lastly, it is recommended that researchers using suction-cup tag technology should aim to collect samples from recovered tags even if the data is outside the intended scope of their work to increase the data available to the wider research community.

## Supporting information

S1 File Step-by-step protocol, also available on protocols.io. (PDF)

S1 Table All samples used in this study. Successful PCR runs are in bold. Mn: humpback whale; NARW: North Atlantic right whale; SBNMS: Stellwagen Bank National Marine Sanctuary; SNE: southern New England; CCB: Cape Cod Bay; NM: not measured.
(DOCX)

S1 Fig Images of the gels supporting all results. (PDF)
