# Supplementary material for: A minimally invasive marine mammal sex determination method using epidermal tissue recovered from suction-cup tags
Source: PLoS One. 2025 May 23;20(5):e0323658. doi: 10.1371/journal.pone.0323658 (PMC12101714; doi:10.1371/journal.pone.0323658)
Supplement: S1 File — (PDF) [file pone.0323658.s001.pdf]

Marine mammal sex determination using epidermal tissue recovered from suction-cup tags

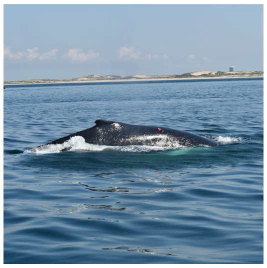

RESERVED DOI:
10.17504/protocols.io.5qpvor42dv4o/v1

Sadie Novak[1], Jacob MJ Linsky[2], kjknapp[1], Susan Parks[1], David Wiley[3], Dana Cusano[1]

[1]Syracuse University; [2]The University of Queensland; [3]Stellwagen Bank National Marine Sanctuary

Dana Cusano
Syracuse University

Protocol Info: Sadie Novak, Jacob MJ Linsky, kjknapp, Susan Parks, David Wiley, Dana Cusano . Marine mammal sex determination using epidermal tissue recovered from suction-cup tags. **Protocol exchange** **https://protocols.io/view/marine-mammal-sex-determination-using-epidermal-ti-cr78v9rw**

Created: March 31, 2023

Last Modified: December 02, 2024

Protocol Integer ID: 79840

Keywords: Polymerase chain reaction, trace DNA, sex determination, marine mammal

Funders Acknowledgement:
Volgenau Foundation
Grant ID: NA
International Fund for Animal Welfare
Grant ID: NA
Bureau of Ocean Energy Management
Grant ID: NA
Stellwagen Bank National Marine Sanctuary
Grant ID: NA

Abstract

For many marine mammal species, minimal sexual dimorphism means that sex cannot be reliably identified through observation. Instead, genetic analysis of sampled skin and blubber are required to determine an individual's sex. Studies using suction cup tags on free-ranging whales may retrieve exfoliated epidermal tissue from the underside of the tag's suction cups, offering a valuable source of genetic information. This study provides a protocol for tissue collection and DNA extraction from recovered tag tissue and describe the accuracy of PCR-based sex determination using these methods.

Image Attribution

Image taken under NMFS permit 18059. Image credit: Laura J. Howes.

Materials

MyTaq Extract-PCR Kit (Meridian Bioscience, cat. no. BIO-21126)
DNEasy Blood and Tissue Kit (Qiagen, cat. no. 69504)
Protocol-specific primers:
Forward SRY: 5' CCC ATG AAC GCA TTC ATT GTG TGG 3'
Reverse SRY: 5' ATT TTA GCC TTC CGA CGA GGT CGA TA 3'
Forward ZFX: 5' ATA ATC ACA TGG AGA GCC ACA AGC T 3'
Reverse ZFX: 5' GCA CTT CTT TGG TAT CTG AGA AAG T 3'

Field Sampling

1 Retrieve the tag from the water. While retrieving the tag, ensure that nothing touches the suction cups with the exception of the net used for retrieval and wear gloves to prevent contamination.

2 Using fresh nitrile gloves, wipe the inside of the suction cups with a sterile swab, avoiding the outside of the suction cup and any other part of the tag. This reduces the chances of obtaining a sample that is contaminated with the genetic material of another animal that may have come into contact with the tagged animal. Note that suctions cups may not have visible skin.

3 Swab two suction cups with one swab, then the other two suction cups with a new sterile swab. Retrieving two samples from the same tag allows for more testing as well as sample security in cases of contamination.

4 Place the swab tip first into a plastic vial, trimming the handle as needed in order to close or screw on the lid.

5 Clearly label the vial with the date and the identifying information that is associated with that specific deployment. Make sure that there is enough information on the label to ensure that all samples are easily and accurately associated with the respective deployment data. This includes, at a minimum, the ID associated with the tag deployment. For example 'bb22_115a', indicating the two letter species ID (bb), julian day (115), and the order of deployment (a, the first tag of the day).

5.1 It is also important to note this information on a datasheet designed to store this data along with other metadata that will be needed later. Of particular importance are:

- Date the sample was collected
- Time the sample was collected
- Location (latitude and longitude) the sample was collected
- Group size/proximity to other animals, as these factors may be a source of contamination

6 Move the vial immediately into a -80 °C freezer if possible. If no -80 °C is available on board the vessel, store on dry ice until the sample can be moved into an appropriate freezer.

7 Repeat steps 3 through 6 for the remaining pair of suction cups.

DNA Extraction

11h 25m 45s

8 **Note**

DNA extraction was performed with a commercially available kit (DNease Blood & Tissue Kit, Qiagen, cat. no. 69504).

To begin DNA extraction, remove swab tip from plastic stick. This can be done by tracing a clean razor blade along the connection point from the swab tip to the swab stick. Transfer the swab tip to
a clean 1.5-mL microcentrifuge tube for each sample.

> **Note**
>
> Be sure to use a clean razor blade for each sample to avoid cross-contamination of any genetic material.

9 Add the amount of Buffer ATL directly to each tube containing swab as directed by the manufacturer's protocol. Pipette solution up and down and over entirety of swab thoroughly, at least 20 times.

10 Add the amount of Proteinase K to each tube containing the swab and previous buffer solution as directed by the manufacturer's protocol. Pipette the solution up and down over the swab to mix. Vortex each sample for 00:00:15 on high to ensure thorough mixing. 15s

11 Incubate samples at 56 °C in a hot water bath for 03:00:00 . 3h

12 Add Buffer AL to each sample as directed by the manufacturer's protocol and mix thoroughly by quickly vortexing for 00:00:15 . Immediately add 200 µL of [M] 96 % (v/v) EtOH and mix again by vortex for 00:00:15 . 30s

13 Pipette solution into the DNeasy Mini spin column and place column in a 2 mL collected tube (included in kit). Incubate samples at Room temperature for 00:05:00 before centrifuging samples at 6000 x g for 00:05:00 . Discard flowthrough and collection tube. Place spin column in a clean 2 mL collection tube (included in kit). 10m

> **Note**
>
> Do not discard flowthrough in this step or any step in the downstream processing into bleach. Chloroform gas will be generated.

14 Add Buffer AW1 to each sample as directed by the manufacturer's protocol and centrifuge samples at 6000 x g for 00:05:00 . Discard the resulting flowthrough and collection tube. Place the spin column in a clean 2 mL collection tube (included in kit). 5m

15 Add Buffer AW2 to each sample as directed by the manufacturer's protocol and centrifuge samples at 17000 x g for 00:03:00 to completely dry the column membrane. Discard the resulting flowthrough and collection tube. Place spin column in a clean 2 mL collection tube (included in kit). 3m

Note

This step is important to ensure no residual ethanol is carried in downstream processing steps of the DNA extraction. If the bottom of the spin column comes in contact with any flowthrough solution, place spin column in a clean 2 mL collection tube and repeat the centrifugation step (17000 x g for 00:03:00)

16 Place the dried spin column into a clean 1.5 mL eppendorf tube and apply 100 µL Buffer AE onto the center of the column membrane. Be sure not to touch the membrane with the pipette tip. Allow the buffer to incubate on the membrane for 00:05:00 at room temperature. Elute DNA by centrifuging the column at 6000 x g for 00:01:00 . Elute DNA from the column again by applying 100 µL Buffer AE onto the center of the column membrane and centrifuging at 6000 x g for 00:01:00 . 7m

Note

The elution step can be performed once with 200 µL of buffer. Eluting DNA step-wise will provide an increased DNA yield.
Do not exceed an elution volume of 200 µL to ensure no flowthrough is refluxed into the bottom of the spin column. This may yield samples with lower purity.

17 Dry DNA samples in a speed vac overnight at room temperature. Re-solubilize DNA in 20 µL of ultrapure water. Measure sample concentration (ng/uL) and purity (260/280 value) using a Nanodrop. 8h

PCR Amplification 33m

18

Note

PCR amplification was performed with OneTaq DNA polymerase (New England BioLabs, cat. no. M0480S) and the standard reaction buffer supplied with the enzyme.

PCR amplification was performed on extracted DNA samples using primers specific to the SRY (sex-determining region Y) and ZFX/ZFY (sex chromosomal zing-finger) genes. Oligonucleotide primers for both of these genes have been validated in other species. Primers were ordered from IDT DNA.
Forward SRY: 5’ CCC ATG AAC GCA TTC ATT GTG TGG 3’
Reverse SRY: 5’ ATT TTA GCC TTC CGA CGA GGT CGA TA 3’
Forward ZFX: 5’ ATA ATC ACA TGG AGA GCC ACA AGC T 3’
Reverse ZFX: 5’ GCA CTT CTT TGG TAT CTG AGA AAG T 3’

19 Prepare the following reaction mixture for each sample of interest in 0.2 mL PCR-grade tubes:

Reagent	Volume/reaction (uL)	Final reaction concentration
OneTaq 5X Standard Buffer	5.0 uL	1X
10 mM dNTPs	0.5 µL	200 µM
20 uM forward ZFX/ZFY primer	0.5 µL	0.4 µM
20 uM reverse ZFX/ZFY primer	0.5 µL	0.4 µM
20 uM forward SRY primer	0.5 µL	0.4 µM
20 uM reverse SRY primer	0.5 µL	0.4 µM
deionized water	15.0 µL	
Template DNA (extracted genomi c DNA)	2.0 µL	
OneTaq DNA polymerase	0.5 µL	2.5 U

Table 1: Reaction components for PCR amplification reaction.

Note

If necessary (to increase band intensity during analysis by gel electrophoresis), the volume of template DNA added to the reaction can be increased. To keep the final volume of the reaction at 25 µL, offset the added volume from the template DNA by subtracting the same volume of deionized water when calculating the reaction components before mixing.

19.1 Amplify DNA fr33magments of interest with 30 cycles of PCR at the following conditions: 33m

Step	Temperature (°C)	Time (seconds)	Number of cycles
y	m		Cycles
Initial Denatur ation	95 °C	180	1
Denaturation	95 °C	15	30
Annealing	55 °C	15	
Extension	72 °C	20	
Final extensio n	72 °C	300	1

Table 2: PCR amplification cycles

Analysis of PCR product by gel electrophoresis

1h 27m

20 The PCR product will be assessed by gel electrophoresis using the known length of the SRY (210-260 bpd) and ZFX/ZFY (442/445). This protocol is written for use with an EasyCast B1 Mini Gel Electrophoresis system (ThermoFisher).

21 Cast a [M] 2 % volume agarose gel dyed with SYBR Safe DNA gel stain.

21.1 Prepare mold by placing gel tray in the buffer chamber of the electrophoresis apparatus so that the gaskets of the tray form a seal with the walls of the chamber. Use a level to adjust the tray position so that the gel is cast evenly.

21.2 Prepare the 2% agarose solution by combining the appropriate mass of agarose with the appropriate volume of 1X tris-acetate EDTA (TAE) buffer (i.e. for a 75 mL agarose gel, combine 1.875 g agarose in 75 mL 1X TAE buffer).

21.3 Melt agarose by microwaving solution in 00:00:15 - 00:00:30 time increments, swirling flask between heatings. 2m

21.4 Allow solution to cool at room temperature for 00:05:00 before adding appropriate volume of SYBR Safe dye to the solution to dilute to 1X SYBR Safe DNA gel stain (i.e. for a 75 mL agarose gel, add 7.5 µL 10,000X SYBR Safe DNA gel stain to solution). 5m

Note

If solution polymerizes in its melting vessel before it is stained and cast, the solid agarose solution can be re-melted as described in step 21.3. If the solid agar was stained and polymerized before casting, the gel can be melted, but should be re-stained to a final concentration of 1X stain.

Note

Other DNA stains such as GelRed or Ethidium Bromide (EtBr) may be used for this step, at appropriate concentrations. SYBRSafe is recommending for its biosafety properties and resulting ease of use.

21.5 Once agarose solution is stained, cast immediately in appropriate gel mold. Place a comb in the mold to create a row of wells. Allow gel to polymerize at Room temperature for 00:20:00 under dark conditions to protect the DNA stain. 20m

22 Re-position gel tray with gel in the buffer chamber of the gel electrophoresis apparatus to the running position (wells should be placed in the chamber so that they are positioned at the negative electrode). Fill the buffer chamber with 1X TAE buffer so that the gel is completely submerged. Remove comb.

23 Dilute running dye in PCR product mixture to a final concentration of 1X xylene cyanol (runs at ~4000 kbp) by adding 4 µL of a 6X xylene cyanol solution to each sample product.

24 Load 2.5 µL of 100 bp DNA molecular weight marker (New England Biolabs, cat. no. NEB#B7025) prepared to contain 1X bromophenol blue running dye into the first well (bromophenol blue runs at ~500 bp). Load 10 µL of the sample(s) in the following wells.

25 Place the lid on the electrophoresis apparatus and ensure that cables are connected to the power supply. Run the gel at 80-100 V under dark conditions. Monitor gel run until the bromophenol blue running dye line has migrated about 1 cm from the midpoint of the gel (~ 00:45:00 - 01:00:00). 1h

26 Once the gel run is complete, resolved DNA fragments may be visualized with a UV transilluminator.

Note

In this protocol, a ChemiDoc imaging system (Bio-Rad) was used to image the resolved PCR products following separation by gel electrophoresis. The blue tray and the protocol pre-programmed in the instrument for use with SYBR Safe DNA stain was used.

Product analysis

27 The expected molecular weight of the PCR product associated with the SRY gene is 210-260 bp and the expected molecular weight of the PCR product associated with the ZFX/ZFY gene 442-445 bp.
The presence of the SRY gene suggests that the DNA sample came from a male individual. Females will not possess this gene, and thus this DNA fragment will not be amplified in DNA extracted from a female. However, absence of this gene may be caused by issues in the experimental workflow, such as an insufficient amount of template DNA added, which would result in a false result. To validate the PCR reaction and minimize the chance a false sexing, DNA is also screened simultaneously for a gene that is present in both male and female individuals. The ZFX/ZFY gene serves as a positive control in this protocol.
An expected profile for DNA extracted from a male individual would show the presence of DNA fragments from both the SRY gene (210-260 bp) and the ZFX/ZFY genes (442-445 bp). An expected profile for DNA extracted from a female individual would show presence of only the DNA fragement from the ZFX/ZFY gene (442-445 bp). A sample that showed presence of only the DNA fragment from the SRY gene would suggest non-specific priming and would be considered an inconclusive result.

Expected result

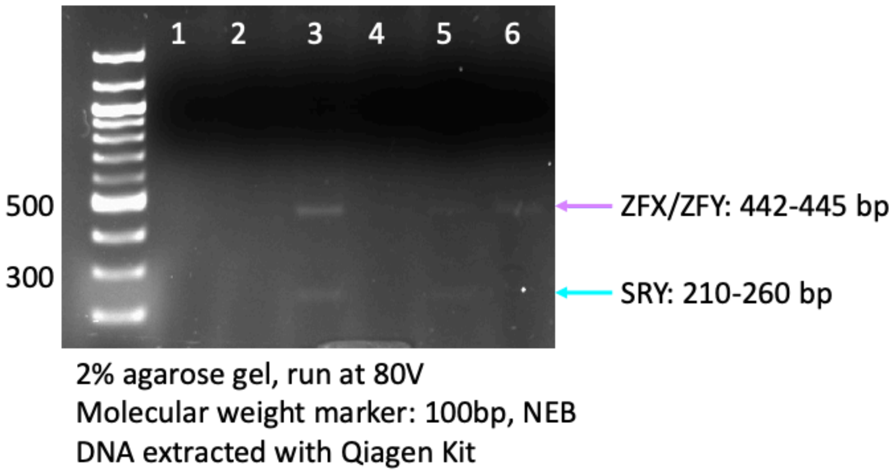

Figure 1: The results of six samples of extracted DNA, amplified following the protocol described above, and resolved via gel electrophoresis. Samples run in lanes 1, 2, and 4 yielded no bands. Samples run in lanes 3 and 5 show an amplicon profile indicative of a male individual, with two bands at 442-445 bp (magenta arrow) and 210-260 bp (cyan arrow). The sample run in lane 6 shows an amplicon profile indicative of a female individual, with one band resolved at 442-445 bp (magenta arrow), only.
