# Supplementary material for: A minimally invasive marine mammal sex determination method using epidermal tissue recovered from suction-cup tags
Source: PLoS One. 2025 May 23;20(5):e0323658. doi: 10.1371/journal.pone.0323658 (PMC12101714; doi:10.1371/journal.pone.0323658)
Supplement: S1 Table — Successful PCR runs are in bold. Mn: humpback whale; NARW: North Atlantic right whale; SBNMS: Stellwagen Bank National Marine Sanctuary; SNE: southern New England; CCB: Cape Cod Bay; NM: not measured. (DOCX) [file pone.0323658.s002.docx]

A minimally invasive marine mammal sex determination method using epidermal tissue recovered from suction-cup tags

**Supplementary Table S1. All samples used in this study. Successful PCR runs are in bold. Mn: humpback whale; NARW: North Atlantic right whale; SBNMS: Stellwagen Bank National Marine Sanctuary; SNE: southern New England; CCB: Cape Cod Bay; NM: not measured.**

| Date | Species | Site | Deployment ID | Verified sex | PCR sex result | Kit | Extraction concentration (ng/µl) | 260/  280 | DNA yield (ng) |
| --- | --- | --- | --- | --- | --- | --- | --- | --- | --- |
| **7/17/2022** | **Humpback** | **SBNMS** | **mn22_198a** | **F** | **F** | **Meridian BioLine** | **NM** | **NM** | **NM** |
| 7/17/2022 | Humpback | SBNMS | mn22_198b | F | No result | Meridian BioLine | NM | NM | NM |
| **7/17/2022** | **Humpback** | **SBNMS** | **mn22_198c** | **M** | **M** | **Meridian BioLine** | **NM** | **NM** | **NM** |
| 7/20/2022 | Humpback | SBNMS | mn22_201a | M | No result | Meridian BioLine | NM | NM | NM |
| 7/20/2022 | Humpback | SBNMS | mn22_201c | M | No result | Meridian BioLine | NM | NM | NM |
| 7/20/2022 | Humpback | SBNMS | mn22_201d | M | No result | Meridian BioLine | NM | NM | NM |
| **7/20/2022** | **Humpback** | **SBNMS** | **mn22_201f** | **F** | **F** | **Meridian BioLine** | **NM** | **NM** | **NM** |
| 7/20/2022 | Humpback | SBNMS | mn22_201g | F | No result | Meridian BioLine | NM | NM | NM |
| 7/20/2022 | Humpback | SBNMS | mn22_201h | M | No result | Meridian BioLine | NM | NM | NM |
| 5/10/2023 | NARW | SNE | eg23_130a | F | No result | Qiagen DNEasy | 15.7 | 1.85 | 31.4 |
| 5/11/2023 | NARW | SNE | eg23_131a | F | No result | Qiagen DNEasy | NM | NM | NM |
| 5/14/2023 | NARW | SNE | eg23_134a (Sample 1) | F | No result | Qiagen DNEasy | 17.1 | NM | 34.2 |
| 5/14/2023 | NARW | SNE | eg23_134a (Sample 2) | F | No result | Qiagen DNEasy | 11.7 | NM | 23.4 |
| **5/14/2023** | **NARW** | **SNE** | **eg23_134b (Sample 1)** | **M** | **M** | **Qiagen DNEasy** | **26.3** | **NM** | **52.6** |
| **5/14/2023** | **NARW** | **SNE** | **eg23_134b (Sample 2)** | **M** | **M** | **Qiagen DNEasy** | **32.4** | **NM** | **64.8** |
| 5/26/2023 | NARW | SNE | eg23_146a | M | No result | Qiagen DNEasy | 11.7 | NM | 23.4 |
| 5/26/2023 | NARW | SNE | eg23_146b | F | No result | Qiagen DNEasy | NM | NM | NM |
| 5/27/2023 | NARW | SNE | eg23_147a | M | No result | Qiagen DNEasy | NM | NM | NM |
| **7/17/2023** | **Humpback** | **SBNMS** | **mn23_198a (Sample 1)** | **F** | **F** | **Meridian BioLine** | **NM** | **NM** | **NM** |
| **7/17/2023** | **Humpback** | **SBNMS** | **mn23_198a (Sample 2)** | **F** | **F** | **Meridian BioLine** | **NM** | **NM** | **NM** |
| 7/18/2023 | Humpback | SBNMS | mn23_199b (Sample 1) | M | No result | Meridian BioLine | NM | NM | NM |
| 7/18/2023 | Humpback | SBNMS | mn23_199b (Sample 2) | M | No result | Meridian BioLine | NM | NM | NM |
| **7/18/2023** | **Humpback** | **SBNMS** | **mn23_199c (Sample 1)** | **F** | **F** | **Meridian BioLine** | **NM** | **NM** | **NM** |
| **7/18/2023** | **Humpback** | **SBNMS** | **mn23_199c (Sample 2)** | **F** | **M** | **Meridian BioLine** | **NM** | **NM** | **NM** |
| **7/19/2023** | **Humpback** | **SBNMS** | **mn23_200a (Sample 1)** | **F** | **F** | **Meridian BioLine** | **NM** | **NM** | **NM** |
| 7/19/2023 | Humpback | SBNMS | mn23_200a (Sample 2) | F | No result | Meridian BioLine | NM | NM | NM |
| 7/19/2023 | Humpback | SBNMS | mn23_200b | M | No result | Meridian BioLine | NM | NM | NM |
| **7/19/2023** | **Humpback** | **SBNMS** | **mn23_200c**  **(Sample 1)** | **F** | **F** | **Meridian BioLine** | **NM** | **NM** | **NM** |
| 7/19/2023 | Humpback | SBNMS | mn23_200c  (Sample 2) | F | No result | Meridian BioLine | NM | NM | NM |
| 7/20/2023 | Humpback | SBNMS | mn23_201a | M | No result | Qiagen DNEasy | 15.8 | 2.24 | 31.6 |
| 7/20/2023 | Humpback | SBNMS | mn23_201c | F | No result | Qiagen DNEasy | 14.2 | 2.51 | 28.4 |
| **7/22/2023** | **Humpback** | **SBNMS** | **mn23_203a** | **M** | **M** | **Qiagen DNEasy** | **39.7** | **2.08** | **79.4** |
| 7/22/2023 | Humpback | SBNMS | mn23_203b (Sample 1) | F | No result | Qiagen DNEasy | 19.2 | 2.59 | 38.4 |
| 7/22/2023 | Humpback | SBNMS | mn23_203b (Sample 2) | F | No result | Meridian BioLine | NM | NM | NM |
| **7/23/2023** | **Humpback** | **SBNMS** | **mn23_204a** | **M** | **M** | **Qiagen DNEasy** | **19.9** | **2.46** | **39.8** |
| **7/23/2023** | **Humpback** | **SBNMS** | **mn23_204b** | **F** | **F** | **Qiagen DNEasy** | **18.7** | **2.66** | **37.4** |
| **7/23/2023** | **Humpback** | **SBNMS** | **mn23_204c** | **F** | **F** | **Qiagen DNEasy** | **27.1** | **2.37** | **54.2** |
| **7/25/2023** | **Humpback** | **SBNMS** | **mn23_206a** | **F** | **F** | **Qiagen DNEasy** | **19.3** | **2.59** | **38.6** |
| **7/25/2023** | **Humpback** | **SBNMS** | **mn23_206b** | **M** | **M** | **Qiagen DNEasy** | **51** | **2.26** | **102** |
| **7/25/2023** | **Humpback** | **SBNMS** | **mn23_206d** | **M** | **M** | **Qiagen DNEasy** | **14.5** | **2.94** | **29** |
| **3/18/2024** | **NARW** | **CCB** | **eg24_078b** | **F** | **F** | **Qiagen DNEasy** | **5.7** | **7.17** | **28.5** |
| **3/27/2024** | **NARW** | **CCB** | **eg24_087a (Sample 1)** | **M** | **M** | **Qiagen DNEasy** | **36.2** | **1.88** | **181** |
| **3/27/2024** | **NARW** | **CCB** | **eg24_087a (Sample 2)** | **M** | **M** | **Qiagen DNEasy** | **16.2** | **2.17** | **81** |
