# Supplementary material for: A minimally invasive marine mammal sex determination method using epidermal tissue recovered from suction-cup tags
Source: PLoS One. 2025 May 23;20(5):e0323658. doi: 10.1371/journal.pone.0323658 (PMC12101714; doi:10.1371/journal.pone.0323658)
Supplement: S1 Fig — (PDF) [file pone.0323658.s003.pdf]

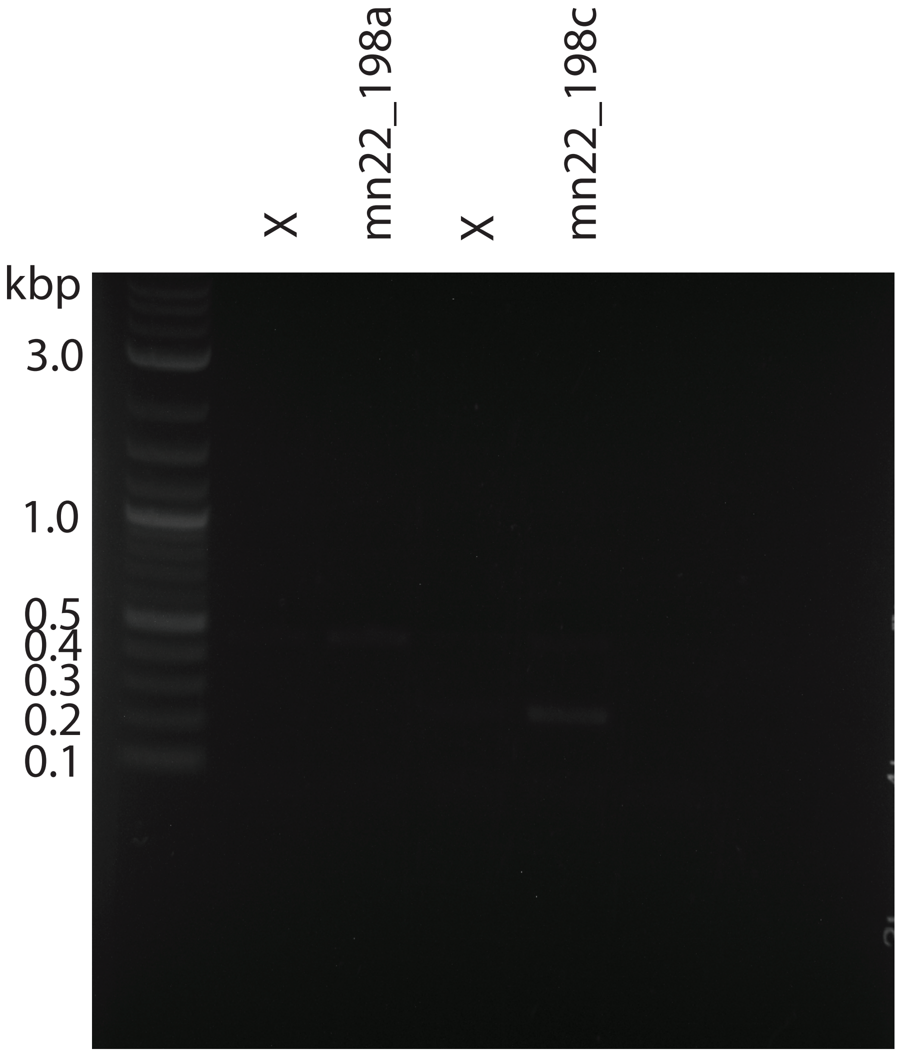

Supplemental Figure 1: Agarose gel showing PCR products amplified from genomic DNA extracted from collected tissue from tissue from the suction cups on acoustic recording tags deployed on the individuals of interest. Genomic DNA was extracted with the DNeasy Blood & Tissue Kit (Qiagen). Following PCR amplification, PCR amplicons were resolved by gel electrophoresis in a 2.0 % agarose DNA gel stained with 1X SYBR Safe DNA gel stain, at 120V. Resulting amplicons were compared against a 1kb molecular weight marker (New England Biolabs). Presence of both the the SRY (210-260 bpd) and ZFX/ZFY (442/445) amplicons indicated a sample derived from a male individual. Presence of the ZFX/ZFY (442/445) amplicon only indicated a sample derived from a female individual.

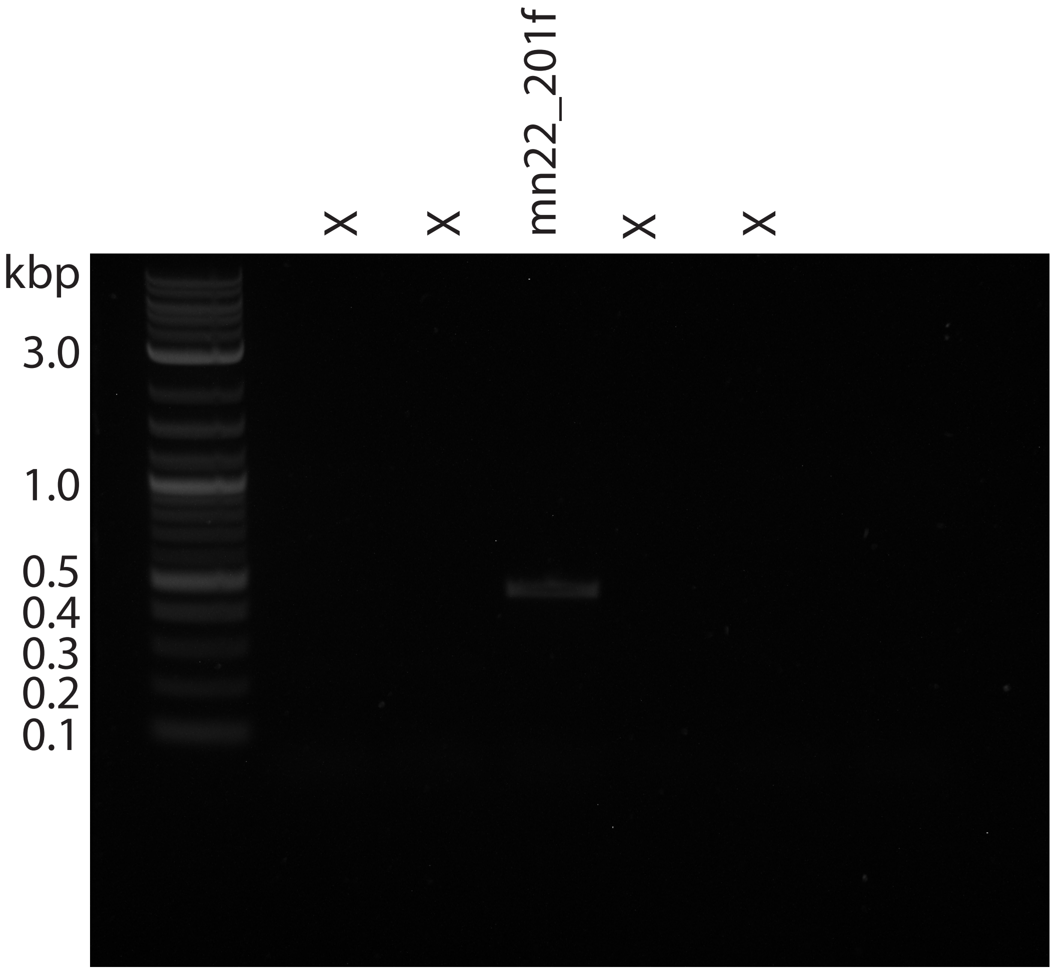

Supplemental Figure 2: Agarose gel showing PCR products amplified from genomic DNA extracted from collected tissue from tissue from the suction cups on acoustic recording tags deployed on the individuals of interest. Genomic DNA was extracted with the DNeasy Blood & Tissue Kit (Qiagen). Following PCR amplification, PCR amplicons were resolved by gel electrophoresis in a 2.0 % agarose DNA gel stained with 1X SYBR Safe DNA gel stain, at 120V. Resulting amplicons were compared against a 1kb molecular weight marker (New England Biolabs). Presence of both the the SRY (210-260 bpd) and ZFX/ZFY (442/445) amplicons indicated a sample derived from a male individual. Presence of the ZFX/ZFY (442/445) amplicon only indicated a sample derived from a female individual.

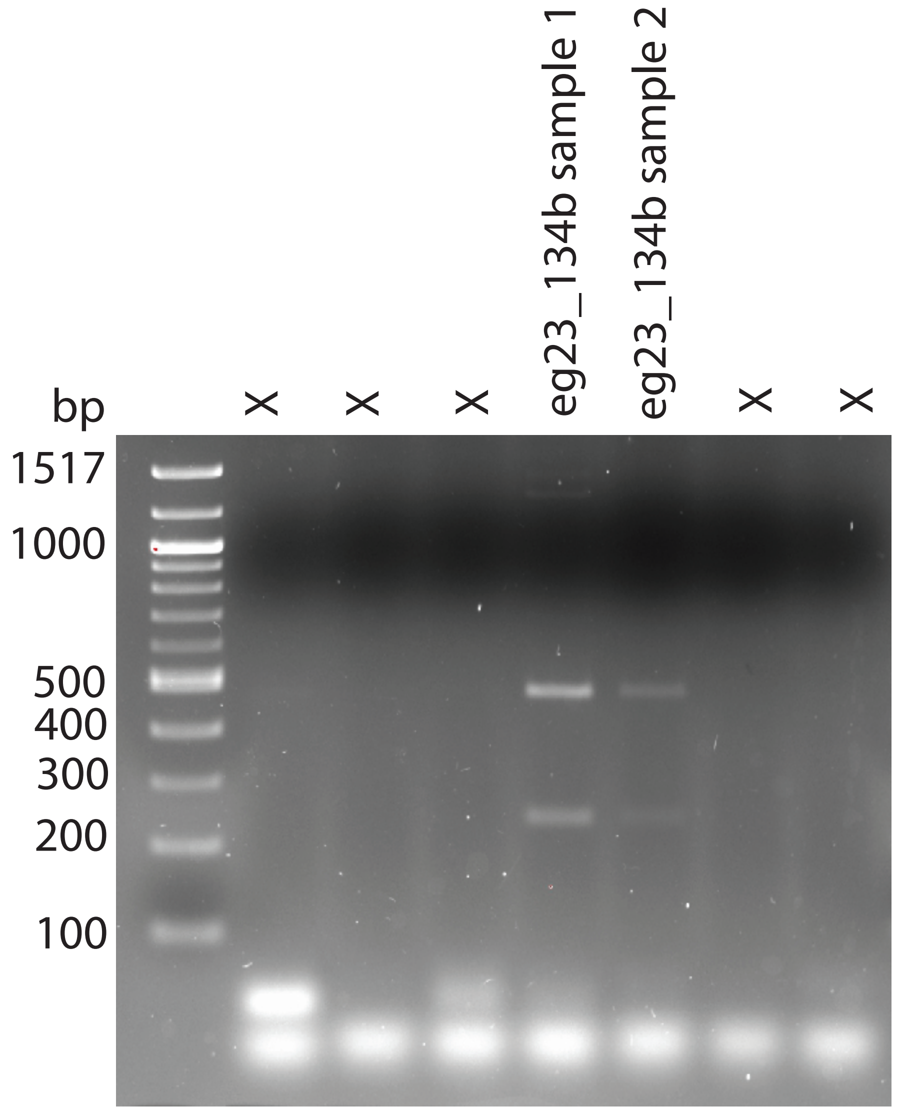

Supplemental Figure 3: Agarose gel showing PCR products amplified from genomic DNA extracted from collected tissue from tissue from the suction cups on acoustic recording tags deployed on the individuals of interest. Genomic DNA was extracted with the DNeasy Blood & Tissue Kit (Qiagen). Following PCR amplification, PCR amplicons were resolved by gel electrophoresis in a 2.0 % agarose DNA gel stained with 1X SYBR Safe DNA gel stain, at 120V. Resulting amplicons were compared against a 100 bp molecular weight marker (New England Biolabs). Presence of both the the SRY (210-260 bpd) and ZFX/ZFY (442/445) amplicons indicated a sample derived from a male individual. Presence of the ZFX/ZFY (442/445) amplicon only indicated a sample derived from a female individual.

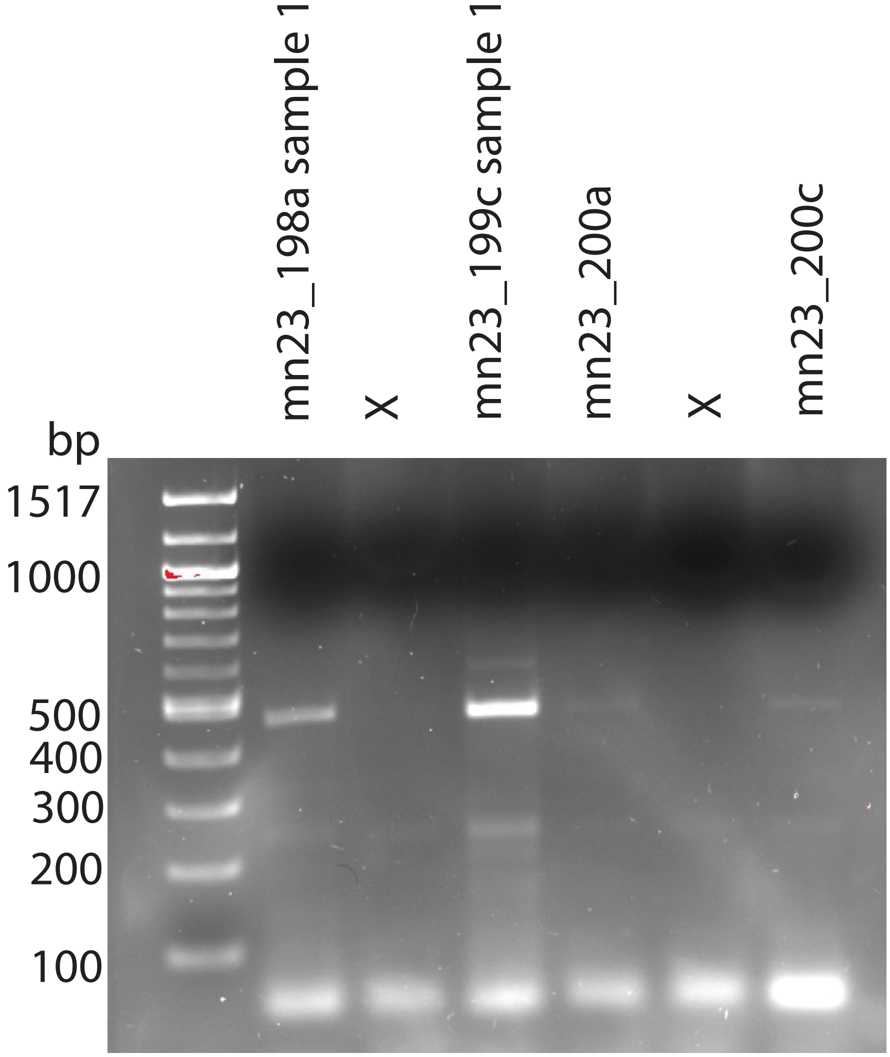

Supplemental Figure 4: Agarose gel showing PCR products amplified from genomic DNA extracted from collected tissue from tissue from the suction cups on acoustic recording tags deployed on the individuals of interest. Genomic DNA was extracted with the DNeasy Blood & Tissue Kit (Qiagen). Following PCR amplification, PCR amplicons were resolved by gel electrophoresis in a 2.0 % agarose DNA gel stained with 1X SYBR Safe DNA gel stain, at 120V. Resulting amplicons were compared against a 100 bp molecular weight marker (New England Biolabs). Presence of both the the SRY (210-260 bpd) and ZFX/ZFY (442/445) amplicons indicated a sample derived from a male individual. Presence of the ZFX/ZFY (442/445) amplicon only indicated a sample derived from a female individual.

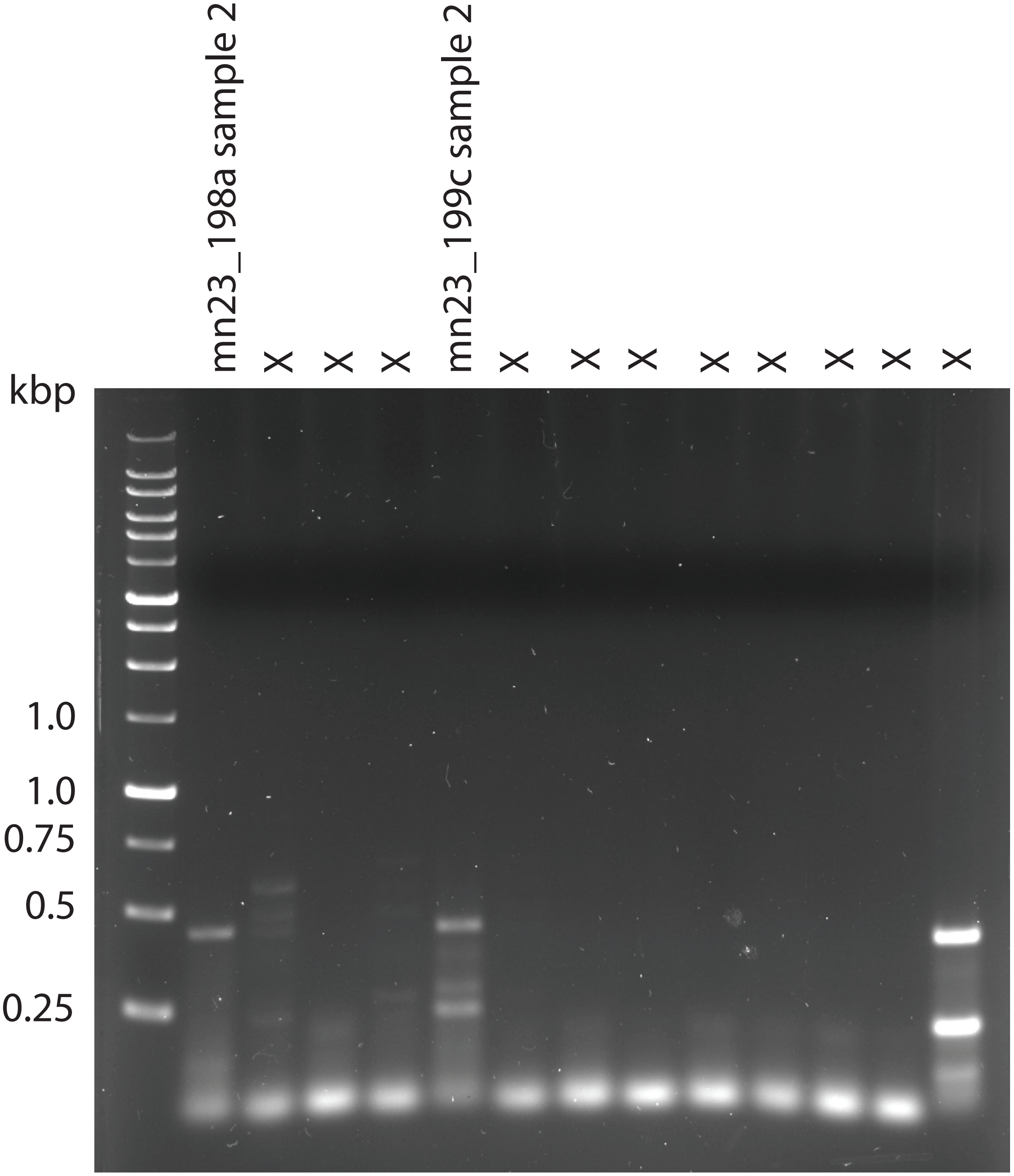

Supplemental Figure 5: Agarose gel showing PCR products amplified from genomic DNA extracted from tissue from the suction cups on acoustic recording tags deployed on the individuals of interest. Genomic DNA was extracted with the MyTaq Extact-PCR kit (Meridian Bioscience). Following PCR amplification, PCR amplicons were resolved by gel electrophoresis in a 0.8 % agarose DNA gel stained with 1X SYBR Safe DNA gel stain, at 120V. Resulting amplicons were compared against a 1 kbp molecular weight marker (ThermoScientific). Presence of both the the SRY (210-260 bpd) and ZFX/ZFY (442/445) amplicons indicated a sample derived from a male individual. Presence of the ZFX/ZFY (442/445) amplicon only indicated a sample derived from a female individual.

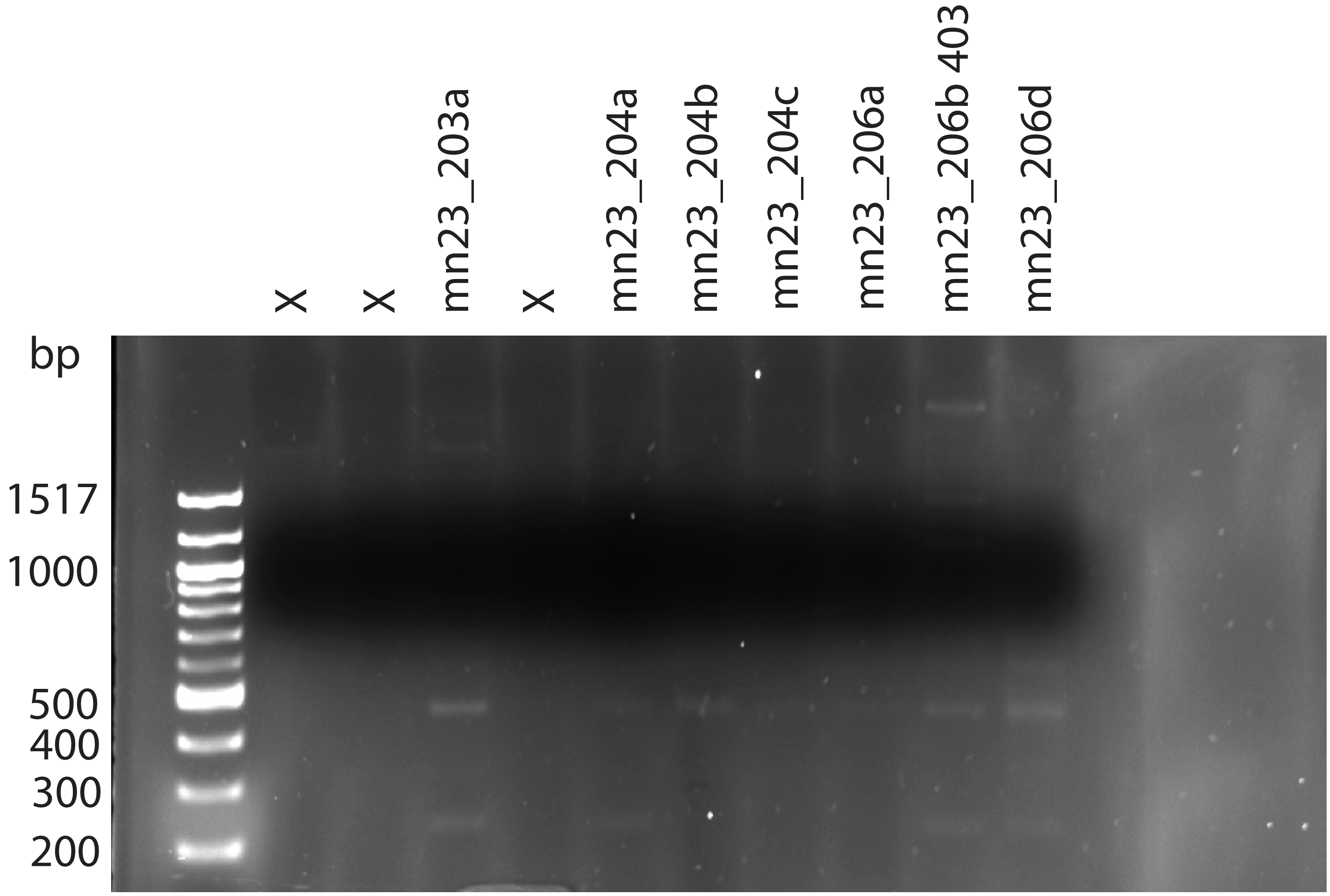

Supplemental Figure 6: Agarose gel showing PCR products amplified from genomic DNA extracted from collected tissue from tissue from the suction cups on acoustic recording tags deployed on the individuals of interest. Genomic DNA was extracted with the DNeasy Blood & Tissue Kit (Qiagen). Following PCR amplification, PCR amplicons were resolved by gel electrophoresis in a 2.0 % agarose DNA gel stained with 1X SYBR Safe DNA gel stain, at 120V. Resulting amplicons were compared against a 100 bp molecular weight marker (New England Biolabs). Presence of both the the SRY (210-260 bpd) and ZFX/ZFY (442/445) amplicons indicated a sample derived from a male individual. Presence of the ZFX/ZFY (442/445) amplicon only indicated a sample derived from a female individual.

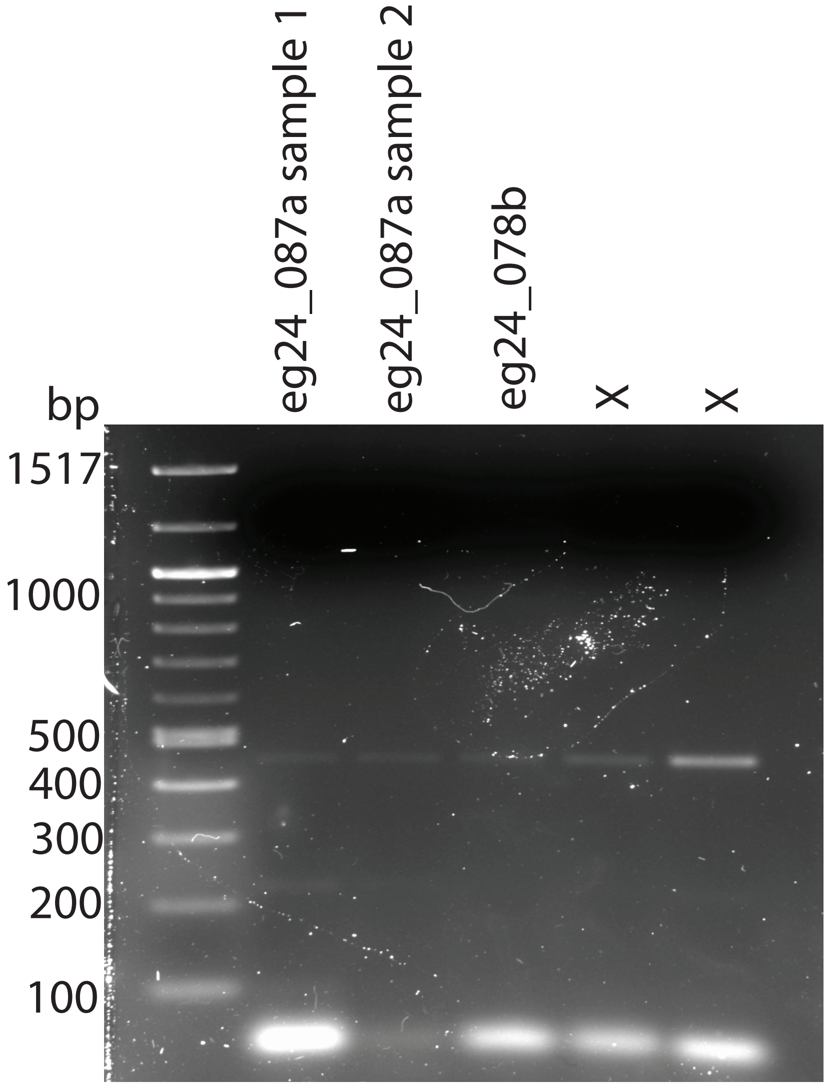

Supplemental Figure 7: Agarose gel showing PCR products amplified from genomic DNA extracted from collected tissue from tissue from the suction cups on acoustic recording tags deployed on the individuals of interest. Genomic DNA was extracted with the DNeasy Blood & Tissue Kit (Qiagen). Following PCR amplification, PCR amplicons were resolved by gel electrophoresis in a 2.0 % agarose DNA gel stained with 1X SYBR Safe DNA gel stain, at 120V. Resulting amplicons were compared against a 100 bp molecular weight marker (New England Biolabs). Presence of both the the SRY (210-260 bpd) and ZFX/ZFY (442/445) amplicons indicated a sample derived from a male individual. Presence of the ZFX/ZFY (442/445) amplicon only indicated a sample derived from a female individual.
